# Biochemical evaluation of Jihua hemostatic suppository in hemorrhagic internal hemorrhoids: Modulation of XPO1, VEGF, and collagen I/III expression and inflammatory biomarkers

**DOI:** 10.5937/jomb0-62625

**Published:** 2026-03-17

**Authors:** Chao Wang, Yajun Kang, Ye Wang, Yujie Liu, Mengli Shi, Xiaoming Liu, Hao Jiao

**Affiliations:** 1 Shijiazhuang Municipal Hospital of TCM, Department of Proctology, Shijiazhuang, China; 2 Shijiazhuang Key Laboratory of Topical Chinese Herbal Formulation R&D, Shijiazhuang, Hebei, China

**Keywords:** Jihua hemostatic suppository, gangtai suppository, hemorrhagic Internal hemorrhoids, exportin-1 (Xpo1), vascular endothelial growth factor (VEGF), collagen I/III, inflammatory biomarkers, biochemical modulation, hemostatske supozitorije Džihua, supozitorije gangtai, hemoragični unutrašnji hemoroidi, eksportin-1 (Xpo1), vaskularni endotelni faktor rasta (VEGF), kolagen I/III, inflamatorni biomarkeri, biohemijska modulacija

## Abstract

**Background:**

Hemorrhagic internal hemorrhoids involve abnormal vascular remodeling, inflammatory activation, and extracellular matrix dysregulation. Exportin-1 (Xpo1), vascular endothelial growth factor (VEGF), and collagen types I and III are key biochemical markers associated with these processes. This study aimed to evaluate the therapeutic efficacy of Jihua Hemostatic Suppository compared with Gangtai Suppository and to investigate their differential effects on biochemical and inflammatory parameters in patients with hemorrhagic internal hemorrhoids.

**Methods:**

A total of 98 patients diagnosed with hemorrhagic internal hemorrhoids were enrolled between January 2024 and January 2025 and randomly divided into two groups (n = 49 each) using the envelope randomization method. The Jihua group received Jihua Hemostatic Suppository, whereas the control group received Gangtai Suppository for two weeks. Serum levels of interleukin-6 (IL-6), tumor necrosis factor-a (TNF-a), and C-reactive protein (CRP) were determined by ELISA. Xpo1 levels were measured by ELISA, VEGF by chemiluminescence assay, and collagen types I and III by the hydroxyproline method. Clinical efficacy, traditional Chinese medicine (TCM) syndrome scores, and adverse reactions were also recorded.

**Results:**

The total effective rate in the Jihua group (93.88% ) was significantly higher than that in the Gangtai group (77.55% ) (c2 = 5.968, P = 0.015). After treatment, the Jihua group showed markedly lower serum IL-6 (70.50 ± 5.82 ng/mL), CRP (23.06 ± 2.71 mg/L), and TNF-a (60.81 ± 5.72 ng/mL) levels compared with the Gangtai group (P &lt; 0.001). Biochemically, Xpo1, VEGF, collagen I, and collagen III concentrations were significantly reduced in the Jihua group (12.31 ± 1.35 ng/mL, 389.64 ± 34.32 pg/mL, 154.39 ± 15.19 pg/L, and 36.13 ± 3.06 mg/L, respectively) compared with the Gangtai group (P &lt; 0.05). The incidence of adverse reactions was lower with Jihua suppository (4.08%) than with Gangtai suppository (20.41% ) (c2 = 4.242, P = 0.039).

**Conclusions:**

Jihua Hemostatic Suppository exerts superior therapeutic and biochemical effects in hemorrhagic internal hemorrhoids compared with Gangtai Suppository. Its efficacy is likely mediated by suppression of the Xpo1/NF-kB inflammatory axis, inhibition of VEGF-driven angiogenesis, and normalization of collagen I/III-dependent extracellular matrix remodeling. These findings highlight the value of integrating biochemical biomarkers into clinical assessment of hemorrhoidal disease and suggest Xpo1, VEGF, and collagen I/III as potential molecular indicators of treatment efficacy.

## Abstract (sr)

**Uvod:**

Hemoragični unutrasnji hemoroidi uključuju abnormalno vaskularno remodeliranje, inflamatornu aktivaciju i disregulaciju ekstracelularnog matriksa. Eksportin-1 (Xpo1), faktor rasta vaskularnog endotela (VEGF) i kolagen tipova I i III su ključni biohemijski markeri povezani sa ovim procesima. Cilj ove studije je bio da se proceni terapijska efikasnost hemostatskih supozitorija Jihua u poređenju sa supozitorijama Gangtai i da se ispitaju njihovi različiti efekti na biohemijske i inflamatorne parametre kod pacijenata sa hemoragičnim unutrašnjim hemoroidima.

**Metode:**

Ukupno 98 pacijenata sa dijagnozom hemoragičnih unutrasnjih hemoroida uključeno je u istraživanje između januara 2024. i januara 2025. godine i nasumično podeljeno u dve grupe (n = 49 svaka) korišćenjem metode randomizacije. Grupa Jihua je primala hemostatske supozitorije Jihua, dok je kontrolna grupa primala supozitorije Gangtai tokom dve nedelje. Serumski nivoi interleukina-6 (IL-6), faktora nekroze tumora-a (TNF-a) i C-reaktivnog proteina (CRP) određeni su ELISA testom. Nivoi Xpo1 su mereni ELISA testom, VEGF hemiluminescentnim testom, a kolagen tipova I i III metodom hidroksiprolina. Takođe su zabeleženi klinička efikasnost, rezultati sindroma tradicionalne kineske medicine (TKM) i neželjene reakcije.

**Rezultati:**

Ukupna efektivna stopa u grupi Jihua (93,88%) bila je značajno viša nego u grupi Gangtai (77,55%) (c2 = 5,968, P = 0,015). Nakon tretmana, grupa Jihua je pokazala znatno niže nivoe serumskog IL-6 (70,50 ± 5,82 ng/mL), CRP (23,06 ± 2,71 mg/L) i TNF-a (60,81 ± 5,72 ng/mL) u poređenju sa grupom Gangtai (P &lt; 0,001). Biohemijski, koncentracije Xpo1, VEGF, kolagen I i kolagen III bile su značajno smanjene u grupi Jihua (12,31 ± 1,35 ng/mL, 389,64 ± 34,32 pg/mL, 154,39 ± 15,19 pg/L i 36,13 ± 3,06 mg/L, respektivno) u poređenju sa grupom Gangtai (P &lt; 0,05). Učestalost neželjenih reakcija bila je niža kod Jihua supozitorija (4,08%) nego kod Gangtai supozitorija (20,41%) (c2 = 4,242, P = 0,039).

**Zaključak:**

Hemostatske supozitorije Džihua pokazuju superiorne terapeutske i biohemijske efekte kod hemoragičnih unutrašnjih hemoroida u poređenju sa supozitorijama Gangtai. Njena efikasnost je verovatno posredovana supresijom inflamatorne ose Xpo1/NF-kB, inhibicijom angioneze izazvane VEGF-om i normalizacijom remodeliranja ekstracelularnog matriksa zavisnog od kolagena I/III. Ovi nalazi ističu vrednost integracije biohemijskih biomarkera u kliničku procenu hemoroidalne bolesti i sugerišu Xpo1, VEGF i kolagen I/III kao potencijalne molekularne indikatore efikasnosti lečenja.

## Introduction

Hemorrhagic internal hemorrhoids represent a prevalent anorectal disorder in colorectal surgery, primarily characterized by rectal bleeding that can substantially impair patients' quality of life. Persistent bleeding, if left untreated, may lead to secondary complications such as iron-deficiency anemia, thereby exerting significant adverse effects on systemic health and overall well-being [1].

A wide range of therapeutic modalities are currently available for hemorrhagic internal hemorrhoids, among which pharmacological therapy remains a cornerstone of management. In particular, rectal suppository administration has gained clinical favor owing to its convenience, targeted local drug delivery, and rapid absorption at the lesion site. *Gangtai Suppository* is one of the most commonly used preparations in this setting; it exerts multiple pharmacodynamic effects, including hemostasis through cooling the blood, detoxification, drying dampness, promoting wound healing, and alleviating pain and swelling, thereby reducing hemorrhoidal symptoms to a certain degree [2]. Nevertheless, clinical observations have indicated that the therapeutic outcomes of *Gangtai Suppository* are sometimes suboptimal, and prolonged use may result in local irritation or diminished efficacy [3]. Consequently, identifying safer and more effective therapeutic alternatives remains of clinical and biochemical importance.

*Jihua Hemostatic Suppository* is a novel traditional Chinese medicine (TCM)-based rectal formulation composed of herbal ingredients with hemostatic, anti-inflammatory, and tissue-regenerative properties. From the perspective of TCM pharmacology, the synergistic actions of its bioactive compounds are thought to enhance mucosal repair, regulate local microcirculation, and promote coagulation, thereby conferring potential therapeutic superiority in the management of hemorrhagic internal hemorrhoids [4].

At the molecular level, several biochemical markers are implicated in the pathological process of hemorrhoidal disease. Exportin 1 (Xpo1) was selected as a biomarker because it regulates nuclear export of proteins involved in inflammation and cell proliferation, which contribute to vascular congestion and mucosal damage in hemorrhoids. Vascular endothelial growth factor (VEGF) was chosen due to its central role in angiogenesis and vascular permeability, directly linked to hemorrhoidal bleeding and hyperplasia. Collagen types I and III were included as they are essential for extracellular matrix integrity and wound healing, with dysregulation leading to impaired tissue repair and persistent hemorrhage [5].

Exportin 1 (Xpo1), a key nuclear export protein, regulates intracellular material transport and has been associated with abnormal cell proliferation and inflammation, both of which may contribute to hemorrhoidal pathogenesis. Vascular endothelial growth factor (VEGF) plays a pivotal role in angiogenesis, and its upregulation is strongly correlated with vascular hyperplasia and hemorrhage within the hemorrhoidal plexus. Furthermore, collagen types I and III, the principal structural components of the extracellular matrix, are essential for maintaining tissue integrity and promoting wound repair. Dysregulated synthesis or degradation of these collagens may lead to impaired healing and persistent bleeding [5].

Therefore, this study aimed to compare the clinical efficacy of *Jihua Hemostatic Suppository* and Gangtai Suppository in patients with hemorrhagic internal hemorrhoids, with a particular focus on the modulation of biochemical parameters including Xpo1, VEGF, and collagen types I and III. By integrating clinical outcomes with biochemical assessments, this study seeks to elucidate the potential molecular mechanisms underlying the hemostatic and reparative effects of *Jihua Hemostatic Suppository*, thereby providing laboratory-based evidence for its clinical application and contributing to a deeper biochemical understanding of hemorrhoidal disease.

## Materials and methods

### General information

A total of 98 patients diagnosed with hemorrhagic internal hemorrhoids and admitted to our hospital between January 2024 and January 2025 were prospectively enrolled. Patients were randomly allocated into either the Jihua group or the Gangtai group using a sealed-envelope randomization method, with 49 cases in each group. A power analysis was conducted to justify the sample size. Assuming a 20% difference in effective rate between groups (based on pilot data), with α = 0.05 and power (1-β) = 0.80, a minimum of 45 patients per group was required. We enrolled 49 per group to account for potential dropouts. Baseline demographic and clinical characteristics, including age, gender distribution, disease duration, and body mass index (BMI), showed no statistically significant differences between the two groups (P > 0.05) (Table 1), ensuring baseline comparability.

**Table 1:** Comparison of baseline characteristics between the two groups (±s, n(%)).

Group	n	Gender<br>(male/female)	Age (years)	Disease duration<br>(years)	BMI<br>(kg/m^2^)
Jihua group	49	26 / 23	40.75 ± 5.51	2.95 ± 0.89	22.06 ± 2.59
Gangtai group	49	28 / 21	39.92 ± 5.87	2.77 ± 0.87	22.19 ± 2.52
t/χ^2^		0.165	0.722	1.012	0.252
P		0.685	0.472	0.314	0.802

### Inclusion and exclusion criteria

### Inclusion criteria:

1. Patients meeting the diagnostic criteria for hemorrhagic internal hemorrhoids [6];
2. Age ≥18 years;
3. Diagnosis confirmed by digital rectal examination and anoscopy, with evident symptoms of rectal bleeding;
4. Provision of written informed consent by the patient and family members.

### Exclusion criteria:

1. Presence of other anorectal diseases such as anal fissure, anal fistula, or rectal polyps;
2. Known allergy to any component of Jihua Hemostatic Suppository or Gangtai Suppository;
3. Pregnant or lactating women;
4. Patients with hematologic disorders or coagulation abnormalities;
5. Patients with psychiatric illness that could impair treatment adherence or cooperation with clinical and laboratory evaluations.

All procedures were conducted in accordance with the Declaration of Helsinki and approved by the institutional ethics committee.

### Treatment protocols

### Gangtai group:

Patients received Gangtai Suppository once daily. Prior to administration, patients were instructed to evacuate the bowels and cleanse the anal region with warm water to maintain local hygiene. After hand hygiene, the suppository was inserted rectally using a sterile finger cot. Patients were positioned either laterally - with the lower leg extended and the upper leg flexed toward the abdomen - or in a natural squatting position. The suppository was inserted approximately 2-3 cm into the anal canal with the conical end forward. Patients remained in a lateral or prone position for 15-20 minutes to facilitate absorption and prevent slippage.

### Jihua group:

Patients in this group were treated with Jihua Hemostatic Suppository once daily following the same procedure. Jihua Hemostatic Suppository is composed of key active ingredients including extracts from Panax notoginseng (for hemostasis and blood circulation), Arnebia euchroma (for anti-inflammation and detoxification), and Rheum officinale (for cooling blood and promoting healing), along with supplementary components like borneol for analgesia and glycerol as a base for rectal administration. These ingredients ensure reproducibility by adhering to standardized TCM extraction protocols. Each suppository was inspected for integrity before use. The lateral position was preferred for optimal exposure of the anus. The suppository was inserted smoothly to a depth of 2-3 cm and retained for 15-20 minutes to ensure adequate dissolution and local absorption.

Both groups continued treatment for two consecutive weeks.

### Evaluation of clinical efficacy

Clinical efficacy was categorized into three grades based on symptomatic improvement and anoscopic findings:

Markedly effective: Complete disappearance of hematochezia; absence or spontaneous reduction of hemorrhoidal prolapse; significant improvement in anal swelling, pain, and discomfort; digital rectal and anoscopic examinations showing marked reduction in hemorrhoidal size and vascular congestion.

Effective: Substantial reduction in frequency and volume of bleeding; partial improvement in prolapse symptoms; relief of anal discomfort and reduction of hemorrhoidal inflammation.

Ineffective: No notable improvement or aggravation of bleeding, prolapse, or pain; persistent or worsened inflammatory signs on examination.

The total effective rate was calculated as the sum of »markedly effective« and »effective« cases divided by the total number of patients.

### Observation indicators

### Inflammatory and biochemical parameters

Peripheral venous blood samples (5 mL) were collected in the morning after an overnight fast, both before and after treatment. Serum was separated by centrifugation at 3,000 rpm for 10 minutes at 4°C and stored at -80°C until analysis.

Inflammatory cytokines: Serum interleukin-6 (IL-6), C-reactive protein (CRP), and tumor necrosis factor-alpha (TNF-α) concentrations were quantified using enzyme-linked immunosorbent assay (ELISA) kits (Shanghai Enzyme-linked Biotechnology Co., Ltd., China) in accordance with the manufacturer's protocols.

### Biochemical indices:

Exportin 1 (Xpo1) levels were determined by quantitative ELISA.

Vascular endothelial growth factor (VEGF) concentrations were measured using a chemiluminescence immunoassay analyzer (Abbott ARCHITECT i2000SR, USA).

Collagen type I and III concentrations were evaluated using the hydroxyproline quantification method, following standard biochemical procedures validated in clinical laboratories.

All assays were performed in duplicate to ensure reproducibility, and intra-assay and inter-assay coefficients of variation were maintained below 10%.

### Traditional Chinese medicine (TCM) syndrome scores

Scores for hematochezia, short dark urine, anal-edge edema, and anal distension were evaluated according to standardized TCM diagnostic criteria before and after treatment.

### Adverse reactions

Incidences of perianal induration, anal fistula, anal-edge edema, and defecation difficulty were monitored throughout the treatment period to assess drug safety and tolerability.

### Statistical analysis

All statistical analyses were conducted using the Statistical Package for the Social Sciences (SPSS) version 26.0 (IBM Corp., Armonk, NY, USA). Quantitative variables were expressed as mean ± standard deviation (χ ± s), and between-group comparisons were performed using the independent-sample t-test. Categorical variables were presented as counts and percentages (n, %) and compared using the chi-square (χ^2^) test. A P-value < 0.05 was considered statistically significant. To improve data integrity, all biochemical data were verified for normal distribution using the Shapiro-Wilk test, and outliers were reassessed using Grubbs' test. Analytical accuracy was further validated by random remeasurement of 10% of samples.

## Results

### Comparison of clinical efficacy between the Jihua and Gangtai Groups

Following two weeks of treatment, the overall therapeutic response differed significantly between the two groups. The Jihua Hemostatic Suppository group achieved a total effective rate of 93.88%, which was markedly higher than that observed in the Gangtai Suppository group (77.55%, χ^2^ = 5.968, P = 0.015). Patients in the Jihua group exhibited faster hemostatic effects, with earlier cessation of hematochezia, greater improvement in mucosal healing as observed under anoscopy, and reduced local congestion and edema. These findings indicate that Jihua suppository achieved a more profound and sustained clinical benefit (Table 2).

**Table 2:** Comparison of clinical efficacy between the two groups (n (%)).

Group	n	Markedly effective	Effective	Ineffective	Total effective rate (%)
Jihua group	49	36 (73.47)	10 (20.41)	3 (6.12)	46 (93.88)
Gangtai group	49	22 (44.90)	16 (32.65)	11 (22.45)	38 (77.55)
χ^2^					4.083
P					0.043

### Comparison of inflammatory factor levels before and after treatment

To evaluate systemic inflammatory modulation, serum concentrations of interleukin-6 (IL-6), C-reactive protein (CRP), and tumor necrosis factor-α (TNF-α) were measured before and after treatment. Both groups showed significant post-treatment reductions in all three inflammatory markers compared with baseline (P < 0.05). However, patients treated with Jihua suppository demonstrated substantially greater decreases in these parameters relative to those receiving Gangtai suppository (Table 3). Specifically, mean post-treatment IL-6, CRP and TNF-α levels in the Jihua group were 70.50 ± 5.82 ng/mL, 23.06 ± 2.71 mg/L, and 60.81 ± 5.72 ng/mL, respectively, compared with 86.63 ± 7.26 ng/mL, 38.35 ± 3.18 mg/L, and 73.35 ± 6.95 ng/mL in the Gangtai group (P < 0.001 for all comparisons).

**Table 3:** Comparison of inflammatory factor levels before and after treatment (χ^2^±s). Note: Compared with before treatment, P < 0.05.

Group	n	IL-6 (ng/mL)		CRP (mg/L)		TNF-α (ng/mL)	
		Before	After	Before	After	Before	After
Jihua group	49	255.65±25.14	70.50±5.82*	97.74±8.28	23.06±2.71*	178.21±15.43	60.81±5.72*
Gangtai group	49	255.14±24.96	86.63±7.26*	97.48±8.66	38.35±3.18*	177.51±14.95	73.35±6.95*
*t*		0.101	13.135	0.152	25.617	0.228	9.752
*P*		0.920	<0.001	0.880	<0.001	0.820	<0.001

These biochemical results suggest that the Jihua preparation exerts a stronger anti-inflammatory and immunomodulatory effect, effectively reducing proinflammatory cytokine release and acute-phase protein synthesis.

### Comparison of traditional Chinese medicine (TCM) syndrome scores

In alignment with biochemical findings, clinical symptom scores based on TCM diagnostic criteria - including hematochezia, short dark urine, anal-edge edema, and anal distension - significantly improved after treatment in both groups (P < 0.05).

However, the Jihua group exhibited more pronounced reductions across all evaluated symptoms, reflecting enhanced clinical relief and superior restoration of anorectal function (Table 4).

**Table 4:** Comparison of TCM syndrome scores before and after treatment (χ^2^±s, points). Note: Compared with before treatment, P < 0.05.

Group	n	Hematochezia		Short dark urine		Anal-edge edema		Anal distension	
		Before	After	Before	After	Before	After	Before	After
Jihua<br>group	49	4.21±0.76	1.18±0.23*	2.56±0.38	0.68±0.16*	2.78±0.33	0.76±0.15*	2.88±0.32	0.96±0.16*
Gangtai<br>group	49	4.13±0.82	2.86±0.36*	2.68±0.34	1.32±0.23*	2.69±0.39	1.62±0.32*	2.85±0.36	1.78±0.28*
*t*		0.501	27.528	1.647	15.990	1.233	17.034	0.436	17.799
*P*		0.618	<0.001	0.103	<0.001	0.221	<0.001	0.664	<0.001

This improvement corresponded with the observed biochemical downregulation of inflammatory markers, indicating that local symptom alleviation may be linked to systemic inflammatory resolution.

### Comparison of biochemical indices: Xpo1, VEGF, collagen type I, and collagen type III

Biochemical assays revealed notable alterations in the molecular markers associated with vascular remodeling and tissue repair.

After treatment, serum Xpo1, VEGF, collagen type I, and collagen type III concentrations were all significantly lower in the Jihua group compared with the Gangtai group (P < 0.05 for all; Table 5).

**Table 5:** Comparison of Xpo1, VEGF, collagen type I, and collagen type III levels before and after treatment (χ^2^±s). Note: Compared with before treatment, P < 0.05.

Group	n	Xpo1 (ng/mL)		VEGF (pg/mL)		Collagen I (pg/L)		Collagen III (μg/L)	
		Before	After	Before	After	Before	After	Before	After
Jihua<br>group	49	35.47±3.10	12.31±1.35*	865.50±54.24	389.64±34.32*	225.48±25.39	154.39±15.19*	75.53±7.49	36.13±3.06*
Gangtai<br>group	49	35.05±3.35	26.56±2.62*	863.21±52.82	508.02±42.26*	223.62±24.87	186.52±18.66*	75.95±7.16	58.06±5.32*
t		0.644	33.844	0.212	15.221	0.366	9.347	0.284	25.013
p		0.521	<0.001	0.833	<0.001	0.715	<0.001	0.777	<0.001

Exportin 1 (Xpo1): Mean serum level decreased from 35.47 ± 3.10 ng/mL to 12.31 ± 1.35 ng/mL in the Jihua group, whereas the reduction in the Gangtai group was less pronounced (26.56 ± 2.62 ng/mL post-treatment). The sharper decline in Xpo1 suggests that Jihua suppository may modulate nuclear export signaling pathways involved in inflammatory gene expression.

Vascular Endothelial Growth Factor (VEGF): VEGF levels decreased from 865.50 ± 54.24 pg/mL to 389.64 ± 34.32 pg/mL in the Jihua group, compared with 508.02 ± 42.26 pg/mL in the Gangtai group. This marked suppression of VEGF indicates improved regulation of pathological angiogenesis and vascular permeability in the hemorrhoidal plexus.

Collagen Type I and III: Both collagen I and III concentrations declined significantly following Jihua treatment (154.39 ± 15.19 pg/L and 36.13 ± 3.06 μg/L, respectively) compared with post-treatment levels in the Gangtai group (186.52 ± 18.66 pg/L and 58.06 ± 5.32 μg/L). These reductions reflect a more balanced extracellular matrix remodeling process and enhanced mucosal tissue recovery.

Taken together, these biochemical outcomes indicate that Jihua suppository not only alleviates inflammation but also modulates the Xpo1-VEGF-collagen regulatory axis, thereby improving vascular stability and promoting effective tissue repair at the molecular level.

### Comparison of adverse reaction incidence

The overall incidence of treatment-related adverse events was significantly lower in the Jihua group (4.08%) than in the Gangtai group (20.41%, χ^2^ = 4.242, P = 0.039) (Table 6).

**Table 6:** Comparison of adverse reaction incidence between groups (n (%)).

Group	n	Perianal<br>induration	Anal<br>fistula	Anal-edge<br>edema	Defecation<br>difficulty	Total incidence<br>(%)
Jihua group	49	1 (2.04)	1 (2.04)	0 (0.00)	0 (0.00)	2 (4.08)
Gangtai group	49	3 (6.12)	3 (6.12)	2 (4.08)	2 (4.08)	10 (20.41)
χ^2^						4.653
P						

Reported adverse reactions included mild perianal induration, transient anal-edge edema, and rare occurrences of anal fistula or defecation difficulty. All adverse effects resolved spontaneously or after symptomatic management, and no serious systemic complications were observed.

The lower frequency of adverse reactions in the Jihua group underscores its favorable biochemical safety profile and superior local tissue compatibility, further supporting its suitability for clinical use.

### Summary of biochemical and clinical correlations

Correlation analysis revealed that reductions in IL-6, TNF-α, and CRP were positively associated with decreases in VEGF and collagen I/III levels (r > 0.6, P < 0.01), suggesting a biochemical linkage between inflammatory suppression and vascular-matrix remodeling. The consistent biochemical trends across inflammatory and structural biomarkers reinforce the hypothesis that Jihua Hemostatic Suppository exerts its therapeutic action through integrated regulation of inflammatory, angiogenic, and extracellular matrix pathways.

## Discussion

Hemorrhagic internal hemorrhoids represent a multifactorial anorectal disorder involving complex interactions between vascular, connective tissue, and inflammatory processes. Anatomically, the rectal venous plexus lacks venous valves, which impairs venous return and predisposes to congestion. The superficial location and thin walls of the upper and lower rectal venous plexuses, surrounded by loose connective tissue, make them vulnerable to dilation and rupture under elevated venous pressure. Increased intra-abdominal pressure during defecation or straining further exacerbates venous engorgement, leading to rupture and bleeding. Additionally, prolonged sitting or standing, chronic constipation, and diarrhea contribute to increased venous pressure and mucosal mechanical stress, resulting in microvascular damage and hemorrhage [7].

Systemic factors also influence disease development. Aging is associated with decreased vascular elasticity and compromised venous wall integrity, heightening the risk of hemorrhoid formation. Similarly, hepatic cirrhosis and portal hypertension can obstruct venous return from the rectal plexus, thereby predisposing to hemorrhoidal swelling and recurrent bleeding [8]. Dietary factors, such as frequent intake of spicy and irritant foods, may aggravate rectal mucosal inflammation and vascular congestion [9]. From a biochemical perspective, these etiological factors collectively promote endothelial dysfunction, excessive angiogenesis, and matrix remodeling, processes that can be monitored through alterations in biomarkers such as VEGF and collagen.

Gangtai Suppository exerts its pharmacological effects through a combination of active components including Sanguisorba officinalis carbonisatus, Galla Chinensis, borneol, berberine hydrochloride, and papaverine hydrochloride. Sanguisorba officinalis carbonisatus acts as a hemostatic agent through its ability to cool the blood and stop bleeding [10]. Galla Chinensis possesses astringent and anti-exudative properties, while borneol provides analgesic and antiedematous effects. Berberine hydrochloride, a known isoquinoline alkaloid, displays antimicrobial and antiinflammatory activity through inhibition of NF-kB signaling and cytokine release, and papaverine hydrochloride induces smooth muscle relaxation, improving microcirculation [11][12]. Despite these effects, Gangtai Suppository provides symptomatic rather than mechanistic relief in severe hemorrhagic hemorrhoids [13], and its long-term use may lead to pharmacological tolerance and decreased efficacy [14].

In contrast, Jihua Hemostatic Suppository represents an improved formulation integrating hemostatic, anti-inflammatory, and tissue-regenerative herbal components that target multiple biochemical pathways. Upon rectal administration, the suppository enables local drug absorption at the lesion site, resulting in direct modulation of vascular and inflammatory mediators. The preparation induces localized vasoconstriction, reduces capillary permeability, and promotes coagulation by activating platelet aggregation and enhancing coagulation factor activity [15][16][17]. Concurrently, its blood-activating and tissue-repairing constituents enhance microcirculatory flow, facilitate oxygen and nutrient delivery, and accelerate collagen remodeling, thereby promoting mucosal regeneration and structural recovery [18].

From a biochemical perspective, the current study demonstrated that treatment with Jihua Hemostatic Suppository significantly reduced serum concentrations of IL-6, CRP and TNF-α, indicating potent anti-inflammatory activity. IL-6 and TNF-α are key cytokines in the inflammatory cascade, activating endothelial cells and promoting VEGF release, which leads to pathological angiogenesis and increased vascular permeability. Their suppression following Jihua treatment suggests a downregulation of the IL-6/TNF-α-VEGF signaling axis, thereby stabilizing vascular endothelium and limiting hemorrhage. The decline in CRP levels further reflects systemic attenuation of acute-phase inflammation and improved mucosal integrity.

Notably, the observed reduction in Xpo1 (Exportin 1) levels provides a new biochemical insight into the pathophysiological modulation achieved by Jihua treatment. Xpo1 mediates the nuclear export of regulatory proteins involved in inflammation and apoptosis, such as NF-kB and p53. Overexpression of Xpo1 may enhance cytoplasmic translocation of NF-kB, leading to excessive transcription of inflammatory cytokines. The significant post-treatment decrease in Xpo1 observed in this study implies that Jihua suppository might inhibit Xpo1-dependent nuclear export, thereby reducing NF-kB activation and cytokine overproduction - a mechanism consistent with the suppressed IL-6 and TNF-α levels observed clinically. This mechanistic link is likely driven by Jihua's active components, such as Panax notoginseng saponins, which inhibit NF-kB pathways (similar to berberine in Gangtai but with enhanced synergy from Rheum officinale), directly suppressing Xpo1-mediated export and leading to reduced inflammatory biomarkers.

Similarly, VEGF, a critical angiogenic factor, plays a central role in vascular proliferation and permeability within the hemorrhoidal plexus. Elevated VEGF levels are characteristic of vascular congestion and microhemorrhage in hemorrhoidal tissue. The pronounced reduction in VEGF following Jihua therapy suggests effective biochemical suppression of pathological angiogenesis. This finding indicates that the herbal formulation not only controls bleeding mechanically but also restores vascular homeostasis at the molecular level. Pharmacologically, Arnebia euchroma in Jihua targets VEGF downregulation through anti-angiogenic naphthoquinones, providing a stronger link to observed reductions compared to Gangtai's milder effects [19][20][21].

The parallel decline in collagen types I and III observed after treatment reflects modulation of extra-cellular matrix (ECM) turnover. Excessive or disorganized collagen deposition contributes to impaired tissue elasticity and delayed healing. By normalizing collagen metabolism, Jihua suppository appears to promote a balanced regenerative response, ensuring optimal tensile strength and structural recovery of the anorectal mucosa. Together, these biochemical adjustments - reduced Xpo1 and VEGF expression coupled with controlled collagen remodeling - underscore the compound's ability to restore vascular and connective tissue integrity through coordinated biochemical regulation.

Clinically, these molecular effects translate into rapid hemostasis, reduced symptom severity, and accelerated mucosal healing. The total effective rate in the Jihua group (93.88%) significantly exceeded that of the Gangtai group (77.55%), aligning with the favorable biochemical profile. The lower incidence of adverse events in the Jihua group (4.08% vs. 20.41%) further supports its superior local tolerance and systemic safety. These outcomes collectively confirm that Jihua suppository not only alleviates clinical symptoms but also exerts a measurable biochemical impact on inflammatory and vascular pathways involved in hemorrhoidal disease.

Taken together, the results of this study demonstrate that Jihua Hemostatic Suppository acts through integrated biochemical mechanisms involving inhibition of the Xpo1/NF-kB inflammatory axis, suppression of VEGF-mediated angiogenesis, and normalization of collagen I/III-dependent ECM remodeling. This multidimensional biochemical regulation offers a mechanistic explanation for its clinical efficacy and reduced recurrence rates.

## Conclusion

In conclusion, the present findings highlight that Jihua Hemostatic Suppository provides not only symptomatic improvement but also measurable biochemical benefits. By modulating inflammatory cytokines, angiogenic factors, and structural proteins, it restores microvascular stability and tissue homeostasis. These results underscore the importance of integrating biochemical biomarker evaluation into clinical studies of hemorrhoidal disease and suggest that Xpo1, VEGF, and collagen I/III may serve as potential laboratory indicators for assessing therapeutic efficacy in future research.

## Dodatak

### Funding

This work was supported by the Research Project on Traditional Chinese Medicine in Hebei Province (2026391).

### Conflict of interest statement

All the authors declare that they have no conflict of interest in this work.
